# Adherence and quality of life in adults and children during 3-years of SLIT treatment with Grazax—a real life study

**DOI:** 10.1038/s41533-018-0072-z

**Published:** 2018-02-12

**Authors:** Hampus Kiotseridis, Peter Arvidsson, Vibeke Backer, Vagn Braendholt, Alf Tunsäter

**Affiliations:** 1 Departments of Respiratory Medicine and Allergology, Skåne University Hospital, Lund University, Lund, Sweden; 2 ALK Nordic, Box 10073, SE-434 21 Kungsbacka, Sweden; 3 Respiratory Research Unit, Bispebjerg University Hospital, Copenhagen, Denmark; 4 Institute of Clinical Medicine, University of Copenhagen, Copenhagen, Denmark; 5 Vagn Braendholt, Department of medicine, Holbæk Sygehus, Holbæk, Denmark

## Abstract

Respiratory allergic disease represents a global health problem, 30% of the population suffers from allergic rhinoconjunctivitis and 20% suffer from asthma. Allergy immunotherapy induce immunological tolerance and thereby modify the response to allergens and sublingual immunotherapy (SLIT) offers the possibility of home administration of allergen therapy, but adherence is more uncertain. The aim of the study was to investigate the adherence with GRAZAX in adults and children ≥ 5 years during three consecutive years of treatment. This was a non-interventional, prospective, observational, multi-center, open-label study to investigate adherence, quality of life, safety and tolerability of GRAZAX in adult and pediatric patients in a real-life setting. During the 3-years study period estimation of adherence was done regularly. Quality of life as well as symptom score was also assessed. In total, 399 patients (236 adults and 163 children) were included in the study. At baseline, 100% suffered from moderate-severe eyes and nose symptoms, and 31% had asthma in the grass pollen season. Overall, 55% completed a 3-years treatment period, whereas 37% stopped before end of study and 8% were lost to follow up. After 3 years, the adherence rate decreased from 98.2% (first month), 93.7% (first year), 93.2% (second year) and 88.9% (third year) and adverse events were the main reason for pre-term termination. The study suggests a good adherence to treatment in a real life setting among the patients finalizing 3-years SLIT therapy. The treatment was effective both on symptoms and HRQL.

## Respiratory allergic disease: potential of self-care immunotherapy revealed

A three-year trial of the allergy immunotherapy GRAZAX shows good adherence and tolerance among adults and children. Respiratory allergic disease is a significant global health burden, with 30 per cent of Europe’s population suffering from hayfever and 20 per cent from allergic asthma. While antihistamines and steroids can treat symptoms successfully, they do not tackle the underlying allergy. Recent progress in immunotherapies such as GRAZAX—a therapy specifically targeting grass pollen allergy—have shown promise. Hampus Kiotseridis at Lund University, Sweden, and co-workers tracked patient adherence and health in 399 adults and children taking GRAZAX over three years. 55 per cent of patients completed the study treatment, with 85 per cent taking GRAZAX six to seven times a week. GRAZAX proved to be well-tolerated overall, effectively tackling symptoms and improving patients’ quality of life.

## Introduction

Respiratory allergic disease has increased dramatically during the last 40 years and it now represents a serious health problem globally.^1–3^ Up to 30% of the European population suffers from allergic rhinoconjunctivitis, 20% suffer from asthma and 15% from allergic skin conditions. By this, allergy is the most frequent chronic disease in Europe and in the western societies as such.^4^ Its negative impact on quality of life, in physical as well as mental domains, is well documented for children as well as adults.^5,6^ Of patients with intermittent allergic rhinitis 79.2% had some impairment of their professional life and 91.8% of their daily life.^7^ Treatment with pharmacotherapy, such as antihistamines and nasal corticosteroids, is effective in reducing symptoms but they do not address the underlying allergy. Nor do they prevent disease progression.^4,8^ Allergy immunotherapy on the other side, induce immunological tolerance and thereby modify the response to allergens and the disease.^9^ Subcutaneous immunotherapy (SCIT) has been used for decades, whereas a newer form of administration, Sublingual Immunotherapy (SLIT), has been launched within the last decade.^10^ Compared to SCIT with 30 or more visits to the physician, treatment with SLIT offers the possibility of home administration, with a reduced use of medical resources. Health economical studies have also proven GRAZAX, grass immunotherapy, to be a cost-effective treatment in the correct patient group.^11^

Adherence has been extensively discussed in all respiratory therapy during last years, as it is documented that around half the prescriptions are not taken as recommended.^12^ If the prescription is appropriate, then this may represent a fail by the patients, the healthcare system and society and the costs are both personal and economic. Accordingly, adherence with AIT as well as other long-term therapies have been questioned when it comes to ordinary clinical practice and the need for collaboration between healthcare and patient is important for a successful outcome.^13^ Immunotherapy is unlike other treatments a therapy with preventive potential, if it is taken as recommended.^14^ Data with GRAZAX in Sweden during one year of treatment showed promising results with acceptable adherence levels.^15^ However, that study duration was only 1 year, and there is no data regarding the whole treatment period of 3 continuous years. In a study based on prescription data of 706 patients receiving pollen SLIT 16% got prescription for 3 years of treatment or more.^16^ In a retrospective anonymous analysis of 123 SLIT patients (trees mix, grass pollen, and house dust mite—drops or tablets) it was found that 61% of the patients fulfilled 3 years of treatment.^17^ Another retrospective study of 142 SLIT-patients (Rhinitis because of allergy to house dust mites) showed that 46% fulfilled their treatment.^18^

Data from big, randomized, double-blind, placebo-controlled trials have shown that GRAZAX clinically significant improves quality of life. However, there is very limited data regarding quality of life in a real-life setting. The aim of the study was to investigate the adherence with GRAZAX treatment in adults and children ≥ 5 years followed for a period of 3 consecutive years. Furthermore, to evaluate how many patients complete GRAZAX treatment and lastly to assess the efficacy according to symptoms and quality of life (HRQL) in a real-life setting.

## Results

In total, 399 patients (236 adults: ≥15–67 years and 163 children: 5–≤15 years) were included in the study. The 50 participating investigators from Denmark and Sweden enrolled between 1–26 patients per investigator. In total 243 (60.9%) from Sweden, 102 (62.6%) children and 141 (59.7%) adults. The corresponding figures for Denmark 156 (39.1%) in total, 61 (37.4%) children and 95 (40.3%) adults. The subject demographic values at baseline are summarized in Table 1.

**Table 1:** Demografic values at baseline and clinical signs of grass allergy and number of single grass/multiallergic

	Total (*n* = 399)	Child (*n* = 163)	Adult (*n* = 236)
Age (yrs)	24.2 (15.1)	10.6 (2.9)	33.6 (12.8)
	17.7 (5.0; 67.3)	10.8 (5.0; 15.2)	34.1 (15.4; 67.3)
	*n* = 399	*n* = 163	*n* = 236
*Gender*			
Male	241 (60.4%)	117 (71.8%)	124 (52.5%)
Female	158 (39.6%)	46 (28.2%)	112 (47.5%)
*Country*			
Sweden	243 (60.9%)	102 (62.6%)	141 (59.7%)
Denmark	156 (39.1%)	61 (37.4%)	95 (40.3%)
*Clinical signs of grass allergy*			
Rhinitis	383 (96.0%)	154 (94.5%)	229 (97.0%)
Asthma	125 (31.3%)	48 (29.4%)	77 (32.6%)
Conjunctivitis	363 (91.0%)	153 (93.9%)	210 (89.0%)
Atopic eczema	40 (10.0%)	14 (8.6%)	26 (11.0%)
Other	86 (21.6%)	26 (16.0%)	60 (25.4%)
*Single grass/multi allergic*			
Single grass	124 (31.1%)	58 (35.6%)	66 (28.0%)
Multi allergic	275 (68.9%)	105 (64.4%)	170 (72.0%)

For categorical variables *n* (%) is presented

### Allergy history

Patients clinical signs of allergy and main reasons for rhinoconjunctivitis are summarized Table 1. It was allowed to fill in a maximum of two main reasons. 68.9% have more than one allergy. The second most occurring allergy, after grass pollen allergy, causing rhinoconjunctivitis for both children and adult is birch pollen allergy. 31.3% had a grass pollen induced asthma. Symptoms from eyes and nose were mainly moderate-severe at baseline. For the patients with asthma, most patients had mild-moderate symptoms. Many patients (57.3%) had general symptoms such as tiredness. Symptomatic medication used at grass pollen season previous to start of GRAZAX treatment were used in almost all patients (99%). Most common were oral antihistamines, used by 96% and nasal steroids (63%), After 3 years of treatment 14% did not use any symptomatic medication (Table 2).

**Table 2:** Symptomatic medication during the study for those fullfilling the treatment

Variable	Visit 1 (*n* = 220)	Visit 3 (*n* = 220)	Visit 4 (*n* = 220)	Visit 5 (*n* = 220)
None	1 (0.5%)	13 (5.9%)	27 (12.3%)	33 (15.0%)
Oral antihistamines	215 (97.7%)	188 (85.5%)	170 (77.3%)	166 (75.5%)
Oral corticosteroids	13 (5.9%)	2 (0.9%)	5 (2.3%)	3 (1.4%)
Oral leukotriene antagonists	9 (4.1%)	6 (2.7%)	6 (2.7%)	5 (2.3%)
Oral other	1 (0.5%)	0 (0.0%)	0 (0.0%)	3 (1.4%)
Nasal antihistamines	38 (17.3%)	27 (12.3%)	15 (6.8%)	17 (7.7%)
Nasal corticosteroids	139 (63.2%)	83 (37.7%)	76 (34.5%)	65 (29.5%)
Nasal chromones	4 (1.8%)	3 (1.4%)	1 (0.5%)	2 (0.9%)
Nasal other	6 (2.7%)	7 (3.2%)	3 (1.4%)	7 (3.2%)
Eye antihistamines	121 (55.0%)	74 (33.6%)	61 (27.7%)	64 (29.1%)
Eye corticosteroids	13 (5.9%)	3 (1.4%)	5 (2.3%)	5 (2.3%)
Eye chromones	35 (15.9%)	10 (4.5%)	14 (6.4%)	13 (5.9%)
Eye other	19 (8.6%)	12 (5.5%)	5 (2.3%)	10 (4.5%)
Inhalation corticosteroids	58 (26.4%)	49 (22.3%)	46 (20.9%)	40 (18.2%)
Inhalation ß2 agonists (short acting)	56 (25.5%)	34 (15.5%)	38 (17.3%)	37 (16.8%)
Inhalation ß2 agonists (long acting)	19 (8.6%)	10 (4.5%)	17 (7.7%)	13 (5.9%)
Inhalation other	1 (0.5%)	7 (3.2%)	6 (2.7%)	7 (3.2%)
Injection corticosteroids	17 (7.7%)	1 (0.5%)	1 (0.5%)	0 (0.0%)
Injection other	1 (0.5%)	1 (0.5%)	0 (0.0%)	0 (0.0%)

For categorical variables *n* (%) is presented

### Adherence

Of 399 enrolled patients 220 (55%) completed 3 years of treatment. Of the children, 69% completed the 3-year treatment period. The most common reason for discontinuation was adverse event. 65% of discontinuation was made shortly after visit two and three. The registered cause of discontinued treatment is shown in Table 3.

**Table 3:** Cause of discontinued treatment that was registered at each visit

Reason by last visit						
Reason for discontinuation	Last visit					
Frequency percent (row percent; column percent)	1	2	3	4	5	Total
Lost to follow up	0	10	10	9	2	31
	0.00	5.59	5.59	5.03	1.12	17.32
	0.00	32.26	32.26	29.03	6.45	
	0.00	16.95	17.24	40.91	12.50	
Bad effect	0	4	6	2	2	14
	0.00	2.23	3.35	1.12	1.12	7.82
	0.00	28.57	42.86	14.29	14.29	
	0.00	6.78	10.34	9.09	12.50	
Poor compliance	2	4	4	2	5	17
	1.12	2.23	2.23	1.12	2.79	9.50
	11.76	23.53	23.53	11.76	29.41	
	8.33	6.78	6.90	9.09	31.25	
Economical reasons	1	1	0	1	1	4
	0.56	0.56	0.00	0.56	0.56	2.23
	25.00	25.00	0.00	25.00	25.00	
	4.17	1.69	0.00	4.55	6.25	
Adverse events	17	26	19	2	4	68
	9.50	14.53	10.61	1.12	2.23	37.99
	25.00	38.24	27.94	2.94	5.88	
	70.83	44.07	32.76	9.09	25.00	
Other	2	6	13	3	2	26
	1.12	3.35	7.26	1.68	1.12	14.53
	7.69	23.08	50.00	11.54	7.69	
	8.33	10.17	22.41	13.64	12.50	
Not known	2	8	6	3	0	19
	1.12	4.47	3.35	1.68	0.00	10.61
	10.53	42.11	31.58	15.79	0.00	
	8.33	13.56	10.34	13.64	0.00	
Total	24	59	58	22	16	179
	13.41	32.96	32.40	12.29	8.94	100.00

Most of the patients, in the intention to treat population, took their Tablets 6–7 times per week. 94.9% after 1 month, 89.3% at visit 3, 91.3% at visit 4, and 81.3% after third grass pollen season. In the pediatric group, the adherence was higher. 97.1% after 1 month, 92.1% after first season, 92.0 after 2 years and 83.1% after 3 years of treatment, Oversight is the most common reason for not taking their tablets as agreed upon.

For patients with 3 years of treatment (per protocol population) the adherence was even higher, starting with 98.2% of adherence during the first month, 93.7% at visit 3, 93.2% at visit 4, and ending with 88.9% after third visit.

68.7% of the patients (ITT) were offered a tool for adherence by the Investigator at visit 1 but a minority of patients used any. Medimemo is the most offered and used from visit 1 until visit 2. If any adherence tools are used later during the treatment this is most often “other”, e.g., reminder on mobile phone.

### Allergy symptoms

**Fig. 1:**
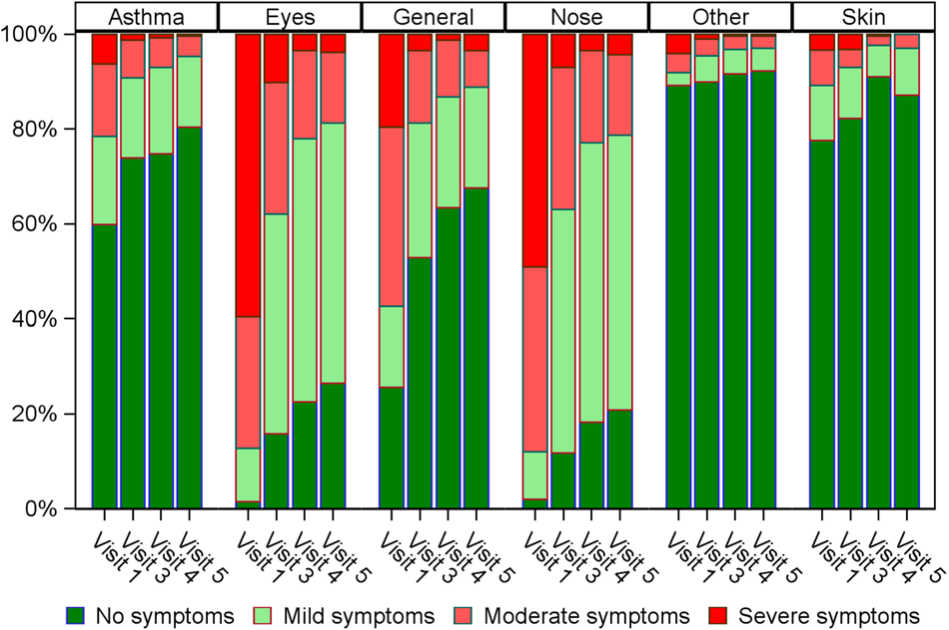
Allergy symptoms. Allergy symptoms during pollen season at baseline (visit 1), after 1 year of treatment (visit 3) and after 2 and 3 years of treatment respectivly (visits 4 and 5). All symtoms improved during treatment including general symptoms

A description of allergy symptoms by visit is presented in Fig. 1. A statistical significant reduction can be seen for all specified symptoms. The largest changes were seen in rhinoconjunctivitis symptoms (eyes and nose) and for general symptoms where tiredness is an example of general symptoms. A reduction in asthma and skin symptoms were also seen. Only few patients specified other symptoms.

The result on the VAS scale question: “How have your allergy symptoms been last GPS during peak” also showed an improvement after first grass pollen season that increases during treatment. A mean at baseline of 7.67 were reduced to 3.86 after first season, 2.9 after second and 2.72 after 3 years of treatment season. In total, a reduction of 5.16 in all 3 years (*p* < 0.01).

**Fig. 2:**
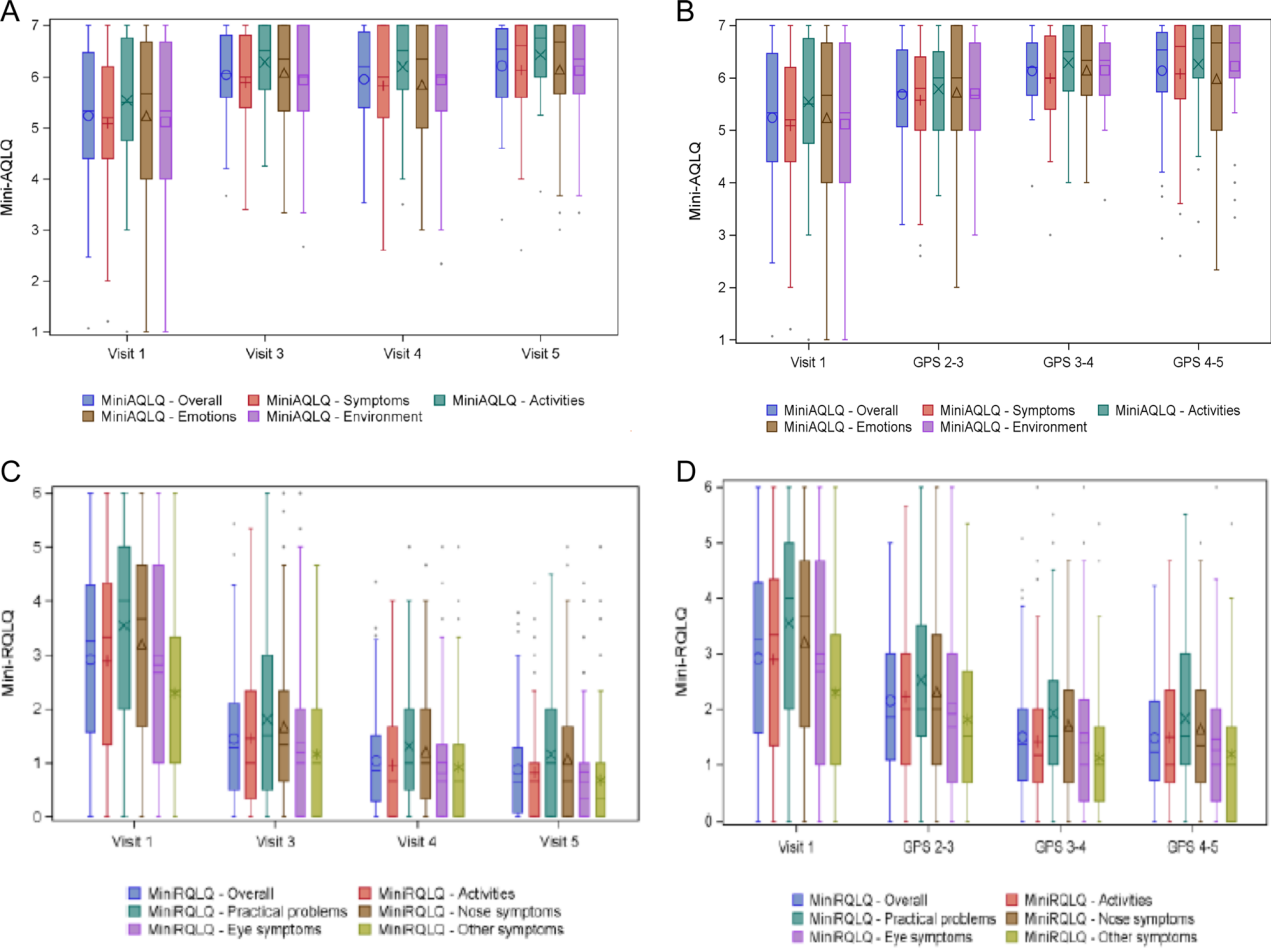
Health related quality of life in adults. The first assessment was done at baseline where patients retrosepectively after pollenseason assessed the symptoms and burden during the preceding pollenseason. Continously the patients assessed the burden during pollenseason and after the pollenseason. In A and B RQLQ was used and in C and D the AQLQ was used. In A and C the assessment was done retrospectively and and in B and D the assessment was done during pollen season

**Fig. 3:**
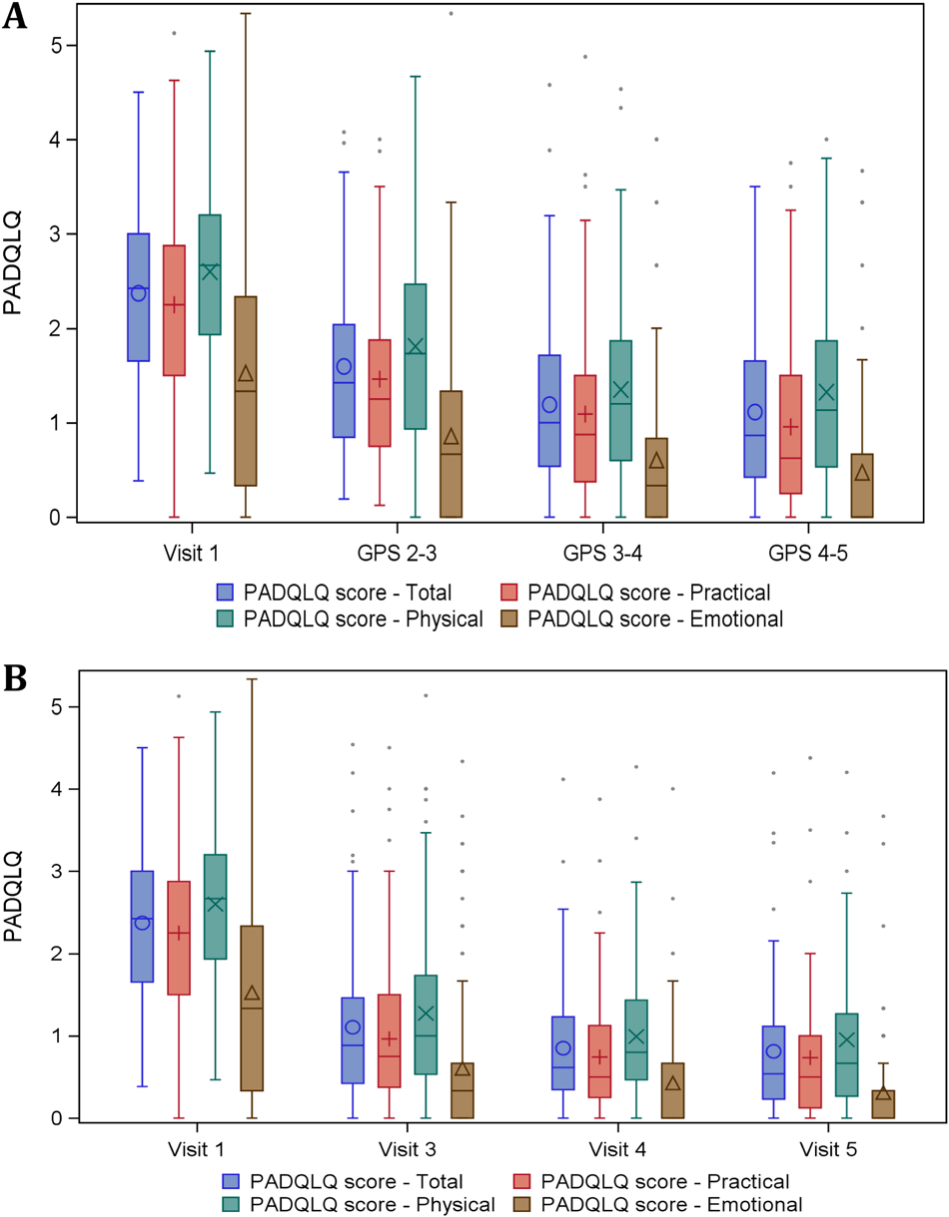
Quality of life PADQLQ subscales and total score by visit and during GPS in children. Assessed during pollenseason (**a**) and as retrospective estimation after pollenseason (**b**)

### Quality of life in adults

Mini-RQLQ showed an improvement when compared to visit 1 (baseline) both for measurements during GPS and at the scheduled visits. Patient scores were lower after GPS than during GPS. The overall score improved from 2.92 at baseline to 1.45 after 1 year, 1.04 after 2 years, and 0.88 after 3 years of treatment. All the domains improved significantly during the 3 years of treatment (*p* < 0.01) (Fig. 2).

As concerns Mini-AQLQ there were improvement when comparing to visit 1 (baseline) both for measurements during GPS and at the scheduled visits between GPS (*p* < 0.01) but not for GPS 2 (*p* = 0.06 and GPS 3 (*p* = 0.49).

There was a good correlation between assessment during pollen season and retrospective assessment although the retrospective assessment showed a tendency to underestimate the problems after the season for PADQLQ, RQLQ (*p* < 0.05) but not the AQLQ. ICC was for PADQLQ; AQLQ and RQLQ 0.56612, 0.73279, and 0.36636, respectively.

### Quality of life in children (aged 5–<15 at start)

PADQLQ showed statistically significant improvement when comparing to visit 1 (baseline) both for measurements during GPS and at the scheduled visits between GPS (Fig. 3).

The total score improved from 2.31 to 1.06 after first year, 0.803 after second year and 0.733 after 3 years of treatment (*p* < 0.01). All domains improved significantly during the treatment period (physical, emotional, and practical). The difference of 1.6 exceeded well beyond 0.4, which is considered as clinical relevant, for the 3-year treatment period

## Discussion

SLIT has been shown in clinical RCT studies to be effective with usually mild side effects. Adherence is an important issue when treating with a tablet taken at home compared to SCIT given at the office. Especially for a long treatment, where it is known that adherence can be challenging. No long-term study has investigated the adherence over 3 years of GRAZAX treatment on patients with rhinitis and/or asthma in ordinary clinical practice. This study therefore adds new valuable real-life data to the existing large pool of data from clinical studies.

The study demonstrated that at least 55% of enrolled patients (220/399) completed 3-years treatment in ordinary clinical practice. Of patients completing treatment, 85% stated they took GRAZAX 6–7 times/week. A recent real-life analysis of SLIT-patients, with rhinitis because of house dust mites allergy, showed that 46% fulfilled treatment.^18^

Adherencerate to SLIT vary a lot in literature. One reason for this could be the different ways to select patients. Some studies in this field are based on prescriptiondata and others on retrospective analysis.^16–18^ In some studies the studypatiens get SLIT with different allergens and in different administrations (drops or tablets).^17^ That makes it difficult to compare the adherence data between studies.

Adherence to prescribed treatment for chronic disease is generally low. Adherence results vary between different studies. For comparison with the results of this study can be mentioned that adherence to antihypertensive treatment is set to 64% in a study from 2008.^19^ The same study shows that adherence to oral antidiabetic treatment is 58% and lipid-lowering medication, 51%. In an asthma study at a pediatric group followed for 2 years the adherence to inhaled corticosteroids was only 50% after 2 years of treatment.^20^ In another study of COPD patients 37% had suboptimal adherence.^21^ Adherence is usually studied for a short time in the context of clinical trials. Rarely in a real-life setting that lasts for 3 years.

According to this real life study, the main reason for not completing 3 years of treatment were adverse events (38% of patients discontinuing treatment). Even if the majority of adverse events were local and mild-moderate, they seem to be bothersome enough for a significant group of patients to stop treatment. An improved practice how to handle adverse events would probably improve adherence. This could be improved by clear and practical information to health care personnel and patients how to handle adverse events. One observation during this study was that although 55% on average completed the 3 years of treatment, the variation in adherence between clinics was great. One explanation to this could be differences between different clinics as concerns interest and literacy to handle patients who undergo this kind of treatment. New initiatives to relieve local side effects in the clinic could be investigated. Early detection of local symptoms that could be treated with symptomatic medication could potentially keep the patients in treatment. In clinical practice experiences with splitting of tablet, moving to other parts of the vestibulum and avoiding swallowing the allergen has proved successful. These methods could be further investigated in an observational study.

The second reason for discontinuing treatment in this trial is lost to follow up (17%), this means that health care has lost contact with the patient. The number of patients continuing treatment in this group is unknown, but hypothetically the number of patients completing treatment could be raised if health care had improved routines for patient follow up.

This study further shows that patient oversight is a minor problem for not taking GRAZAX. Only a minority, 5%, claimed to take their GRAZAX tablet 3 times a week or less. Since intake of tablets were estimated by patients, as this study is not an interventional trial, the information may be biased. Nevertheless, the study design with regular contact to health care seems to have a positive effect on adherence, probably due to increased understanding from the patient why there is a need to be compliant with treatment.

The reasons for not completing the 3-year treatment stated by the patients in this study are in contrast to a recent real-life analysis showing the reasons for premature cessation of treatment to be inability to reach the patient (25%), ineffectiveness of treatment (24%) and the long course (18%).^18^ In “EAACI Guidelines on Allergen Immunotherapy: Allergic rhinoconjunctivitis” the authors found in their review that reported adherence varied from 18 to 90%.^22^ The adherence was better in clinical studies compared to real-life surveys. In that perspectiv our figures for adherence seems to be very good. A reason for this could be that the treatment was carried out in hospitals and in a specialist reception where the routines with patient education and follow ups could be expected to be well anchored.

More children (69%) then adults (45%) remained in treatment during the study. One reason for this outcome could be that caregivers, due to concern about children’s health, gives a reminder to complete treatment. This however, is speculation and needs further investigation.

Health care is good in offering patients different adherence tools, 69% were offered a tool at start of treatment. Patients generally remember to take their GRAZAX as prescribed and usage of adherence tools seems to have a minor effect according to this study since only 33–37% uses any tool during treatment. The same result is previously shown in a one year observational study performed in Northern Europe.^23^ In the study patients were divided in two groups, one using an electronic adherence device (Memozax) and the other group without any device. The adherence was good but no difference was seen between the two groups.

Data from randomized, double-blind, placebo-controlled studies have shown that GRAZAX clinically significant improves quality of life.^23^ However, there is very limited data regarding quality of life in a real-life setting. In this study, the HRQL was improved both in adults and in children. Not only was the total score improved but also the different domains (physical, practical, and emotional).

In the light of allergic disease seen as a systemic disease it seems important to assess also the non-organ specific manifestations. In the present study, the most marked impairment shown was in the physical functioning. Rhinitis is known to affect nocturnal sleep and daytime sleepiness. The impaired sleep may be related to nasal congestion.^24^ A good sleep is of great importance because lack of sleep has consequences both for social functioning and school performance.^25^ We found that not only was physical functioning affected but also the emotional domain was affected. It has been suggested that these effect can have long term effect of the acquisition of personality and health behaviors.^26^ In the study, different QoL questionnaires were used for adults (mini RQLQ, mini AQLQ) and children (PADQLQ) to evaluate QoL at baseline, during and after grass pollen seasons during the study. The reason for choosing this way of evaluation was first to follow the patients during the treatment but also to see if the patients’ retrospective estimation of symptoms and quality of life differs from the estimation during pollen season. Although the AQLQ, RQLQ are validated for symtoms during the last week, in clinical practice the patients are not seldom assessed after the the season and we have before during the validation of the Swedish version of the PADQLQ found good correlation between the assessment during and after the pollenseason. The result showed a good correlation between estimation during pollen season and retrospective estimation after season. although there was a small tendency to underestimate the problems after the season.^27^ Others have also found a fair correlation, but a small tendency for overestimation,^28^ In that study, they used the VDS-4 rating scale 0–3(no, mild, moderate, and severe), for symptom registration and the patients mainly had mild allergic disease wich could affect the result. if QoL is measured after season doctors should bear in mind that the symptoms can be both over and underestimated.

Also, patients’ assessment of allergy symptoms was performed at baseline and during each season. The following symptoms were evaluated: asthma, eye symptoms, general symptoms, nose symptoms, skin symptoms and other. Significant changes from baseline were seen in 5 of the 6 groups. The group not showing any difference was the group other, however only few patients registered “other” symptoms. From a clinical point of view the biggest improvements are as expected in the groups’ eyes, nose and general symptoms. It was clearly seen that the majority of patient moves from moderate-severe symptoms to moderate-mild symptoms, which confirms data from clinical studies.

The positive treatment result is also supported via VAS measurements. Patients compared their overall allergy symptoms during pollen peak over the different seasons and highly significant values were seen. Compared to the severity gradings by Bousquet and coworkers,^29^ the studypatients symptoms seems to be moderate/severe at baseline and mild already after one year. The limit values for MID are met.

However, since no pollen counts were made, a seasonal variation of pollen count affecting the result can not be excluded.

The benefits of SLIT with tablets in form of reduction of symptoms and medication are in line with the systematic reviews concerning the effects of AIT by Dhami et al.^30,31^ They however included also subcutaneous therapy, and also treatment with different sorts of allergens.

## Conclusions

The study demonstrated a good adherence to treatment in a real-life setting. 55% of enrolled patients and 70% of children completed 3 years treatment in ordinary clinical practice where 85% took GRAZAX 6–7 times/week.

The treatment was well tolerated and effective both on symptoms and HRQL.

## Material and methods

### Overall study design

This was a non-interventional, prospective, observational, multi-center, open-label study to investigate adherence, quality of life, safety and tolerability of GRAZAX in adult and pediatric patients in a real-life setting in Sweden and Denmark. That means, the investigator did not affect the selection of patients. They had already been assessed prior to the study, according to clinical practice in the Nordic countries, to be suitable for obtaining AIT.

A desition for Grazax treatment on clinical indication was a prerequisite for the inclusion in the study.

The assessment was done, after evaluation of the symptoms of rhinitis and/or asthma and positive outcome of the test for specific IgE of grass pollen. In addition to questionnaires, they have also not been subjected to any form of intervention beyond clinical practice. The patients did not undergo any training or advice in addition to that given to all patients receiving this treatment in the medical care. This is a single study, not part of or a continuation of an earlier one.

The investigation took place in secondary care units, two allergy clinics in hospitals in Denmark (Copenhagen and Hilleröd) and one Specialist reception for children Sweden (Lund).

The study was initiated in 2011 and the patient were followed up to 36 months, i.e., as long as they were treated with GRAZAX.

When starting a subject on GRAZAX, the investigator planned 5–6 patient visits with approximately 1–12 months in between, according to clinical practice (see Table 4). If visit 5 (i.e., after third GPS) coincided with fulfilled 3 years of GRAZAX treatment, contact 5 became the final visit. However, if the patients started her/his GRAZAX treatment during springtime 2012, treatment did not finish until springtime 2015, which consequently required a sixth visit.

**Table 4:** Study design

Visit 1	Visit 2	Visit 3	Visit 4	Visit 5	Visit 6
Screening and first GRAZAX administration. QoL questionnaire	Follow up adherence, satisfaction and safety. (Phone call optional)	Follow up adherence, satisfaction and safety QoL questionnaire	Follow up adherence, satisfaction and safety QoL questionnaire	Follow up adherence, satisfaction and safety. QoL questionnaire	Follow up adherence, satisfaction and safety
Initiation of study	App. 1 month after visit 1	After first GPS	After second GPS	After third GPS (termination of study if reached 3 yrs treatment)	Final visit, end of study

The assignment of GRAZAX to a patient was not decided in advance by the protocol but was done according to clinical practice. Hence, the decision to prescribe GRAZAX was separate from the decision to include the patient in the study. So, the inclusion criteria for this study is that the doctor already have decided Grazax treatment on clinical indication.

All procedures in this study were in accordance with the 1964 Helsinki declaration. The study was approved by the ethics committee in Lund (2011/508) and patient consent was obtained prior to the start of the study.

### Adherence estimate

At every visit during treatment, patients were asked to estimate how many tablets he/she did take on average since last visit. If not taking the tablet the patients were asked about main reason. Hence—this gave us an oportunity to find out not only the level of adherence but also the reasons for non-adherence. Although this type of subjective measurement has the risk of overestimating adherence it has the advantage of being easy to use and does not interfer much in a real-life setting.^18^

The patients were also asked whether they have used any tools (e.g., SMS or Medimemo) in order to remember to take the GRAZAX tablet. The investigator registered if any tools to help remembering taking the GRAZAX tablet was offered or not. No objective measurement of adherence was used in this study.

### Quality of life (questionnaires and Visual Analogue Scale, VAS)

Quality of life was assessed for the adults by the well-established questionnaires mini RQLQ (Rhinitis quality of life questionnaire) and mini AQLQ (Asthma quality of life questionnaire),^32,33^ designed for assessments of disease specific quality of life in adults for patients with rhinitis and asthma. Both are translated and validated in Swedish and Danish according to guidelines. For both questionnaires the minimal important difference (MID) is considered to be 0.5.

For children, the PADQLQ (Pediatric allergic disease quality of life questionnaire) was used.

This questionnaire has been developed for assessing the multisystem effects of allergic disease on HRQL and takes into account aspects from eyes, nose, lungs, skin, emotions, and everyday activity.^27,34^ It has been shown to have a good cross-sectional and longitudinal validity. It has also been shown to correlate well with allergic inflammation and allergenic load. The Swedish version also showed good reliability for retrospective assessment of the QoL during pollen season.^6^ A difference between repeated measurements of 0.4 is considered clinical relevant.

The PADQLQ can discriminate between children with different degrees of impairment and it has also been shown to be a sensitive marker for change which makes it a good instrument for evaluation of therapy. The questionnaire consists of 26 questions and is self-administered by the children themselves. It takes less than 10 min to answer.

A VAS-scale with a 100 mm long scale, from “not bothersome” to “extremely bothersome” was used. This type of VAS-scale has been proven to be a useful tool in rhinitis as well as in asthma.^29,35^ Bousquet showed that patients with a VAS of under 5 cm could be classified as mild rhinitis (negative predictive value: 93.5%) and those with a VAS of over 6 cm as moderate/severe_ rhinitis (positive predictive value: 73.6%).^29^ All patients assessed the following question:” How have your allergy symptoms been last GPS (Grass pollen season) during peak. This global question includes all allergy symptoms—also asthma. The MID with VAS in rhinitis is by Demoly and coworkers calculated to 23 mm in patients with mild persistent and intermittent allergic rhinitis(AR) and 22 mm in patients with moderate to severe persistent and intermittent AR.^36^ Because uncontrolled asthma is a contraindication to AIT, the study group contains no uncontrolled asthmatics as judged by the researchdoctor and specific asthma questions are therefor not presented.

Allergy symptoms were graded according to the following:

- mild: transient symptoms, no interference with the patient’s daily activities
- moderate: marked symptoms, moderate interference with the patient’s daily activities
- severe: considerable interference with the patient’s daily activities, unacceptable.

At final visit patient and investigator were asked how they experienced GRAZAX treatment: very satisfied, satisfied, dissatisfied or very dissatisfied.

### Statistical methods

All measured variables were summarized by descriptive statistics by visit and change from start of study to each visit if appropriate. The Population, intention to treat or per protocol, is specified. All statistical analysis was nonparametric. Changes over time within total study group and subgroups were analyzed with Wilcoxon Signed Rank test for continuous variables and with Sign test for ordered categorical variables and dichotomous variables.

For comparison between two groups Mann–Whitney *U*-test was used for continuous variables, Mantel–Haenszel Chi-square test for ordered categorical variables, Fisher exact test for dichotomous variables and Pearson Chi-square test for non-ordered categorical variables. Spearman correlation coefficient was used for all correlation analyses. All significance tests were two-sided and conducted at the 5% significance level.

Figures over continuous variables over visits and change in continuous variable from baseline were given as box-plots. Figures over ordered categorical variables and dichotomous variables over visits and change in in these types of variables from baseline were given as bar-charts.

The statistical analyses were performed in SAS 9.3 for Windows (SAS Institute Inc., Cary, NC, USA).

All data are available from the authors.

### Data availability

The datasets generated during and/or analysed during the current study are available from the corresponding author on reasonable request.

### Ethical approval

All procedures in this study were in accordance with the 1964 Helsinki declaration (and its amendments), and the study was approved by the ethics committee, Lund University (nr 2011/508).
